# Editorial: Public health in Africa: role of nutrition and environment

**DOI:** 10.3389/fpubh.2024.1539380

**Published:** 2025-01-23

**Authors:** Guglielmo M. Trovato, Saverio Stranges, A. Kofi Amegah

**Affiliations:** ^1^European Medical Association (EMA), Brussels, Belgium; ^2^Western University (UWO), London, ON, Canada; ^3^University of Naples Federico II, Naples, Italy; ^4^University of Cape Coast, Cape Coast, Ghana

**Keywords:** environment, nutrition, exposome, ultrasound, health literacy, health inequities, Africa, Europe

Many African countries work strongly to protect and improve the health of their populations and to face multiple disease burdens, which include endemic infectious diseases and non-communicable chronic diseases. The main challenges for public health concern the availability of drinking water and its safety, the accessibility and usability of basic sanitation services, urban settlements exposed to a continuous worsening of air pollution, the methods of conservation, processing and cooking food in clean conditions, the ever-increasing small-scale mining activities, the two faces of malnutrition, that is, over- and under-nutrition, and food and nutrition uncertainty and insecurity (1). These challenges are driven by rapid epidemiological, nutritional and socio-economic changes, by demographic transition, population growth, poverty and inequities with socio-economic deprivation, poor governance, corruption, insufficient political will, conflicts and political upheavals. We are witnessing an increasing burden of cardiovascular, respiratory and other chronic diseases, high prevalence of child malnutrition, unfavorable pregnancy and childbirth outcomes, and vulnerability to infectious diseases. Socio-environmental and nutrition policies and actions are urgently needed to mitigate the multiple burdens of disease and the public health challenges on the continent (2). Procedures and strategies to make medical interventions more effective are important, using reliable non-invasive procedures sustainable even in limited-resources subsets. The most concerning threats in Africa include air pollution (domestic and environmental), water pollution, inadequate or non-existent sanitation services, thermal stress, increasingly limited green spaces, contamination from heavy metals, burning of electronic waste, use of pesticides, climate change, poor child nutrition practices, maternal underweight, adulterated and contaminated foods, increased dietary intake of saturated fats, increased consumption of sugars and salt and decreased dietary fiber intake.

Focusing on early exposures in the long term, the life-long perspective has policies implications, i.e., it calls for greater investment in various forms of capital (social, economic, cultural) in people's early years (3). The research studies in this Research Topic attempt to provide some evidence to engage communities and populations affected by these exposures, with the aim of influencing public health policy and programmatic decisions on the continent or specific regions. Although a clear frontier between evidence and lack of evidence in medicine very often has blurred boundaries, and dichotomized confusion and ambiguity can have serious consequences, efforts are needed to seek and provide the best possible evidence (4). Universal health coverage can be fostered through digital solutions, particularly those relevant to pandemic preparedness and response (5). The programs and the use of digital solutions will help strengthen primary healthcare and develop a joint digital health strategy in line with regional needs. This Research Topic of articles has been able to gather significant contributions. These published studies focus on critical and sometimes neglected aspects of healthcare with links to the economy in Africa. These studies help clarify important cultural issues and provide relevant insights.

The first contribution is “It is unbearable to breathe here: air quality, open incineration, and misinformation in Blantyre, Malawi”. Understanding the risk is very limited in all the populations and the groups observed: the poor air quality caused by the burning of waste inside the hospital was perceived as a problem, although there were significant differences between respondents over their understanding of potential impacts, the effectiveness of various coping mechanisms, and their problematizations linked to the burning of specific waste materials. For most, going home and leaving the hospital grounds was the only way to get some relief, although for patients who couldn't leave, staying and enduring was the only option. For more vulnerable populations, especially those with respiratory disease, such as tuberculosis, asthma or other chronic disease, “it could be a matter of life or death” (Tilley et al.).

The second contribution is focused on very critical weaknesses of many health systems and professionals worldwide, particularly severe in limited resources subsets such as in Africa. The article raises the fundamental issue of the educational tools in this model of teaching and training, extremely relevant also in several other fields. Ultrasound is a complex skill to teach and learn. The ability to obtain images through the movement and angle of the probe and the expertise to interpret the images thus produced are crucial to managing patient problems. This procedure requires the refinement of complex visual perception and the implementation of demanding psychomotor skills: it requires personalized and differently extended periods of practical training. The procedure is extremely important for timely and sustainable clinical diagnosis on point-of-care sites and without the need for more complex infrastructure and facilities. The opportunity of allowing the diagnosis of so many diseases related also to environmental factors and bad nutrition is extremely valuable. This is true for any organ disease, also in obstetrics and in pediatrics. Its development and support have the potential to improve greatly and at acceptable costs the quality of health care (Abbattista et al.).

The third research article reports that most children in Côte d'Ivoire, Niger and Senegal are not fed an adequately diversified diet, which depends on an excess of basic starches and has a lower intake of high-quality protein sources (Janmohamed et al.).

The fourth article is a systematic review discussing how good dietary practice is lower than actual knowledge advice and that low are the favorable attitude toward it. They are influenced by distinct sociodemographic variables, income, knowledge and information, beliefs, attitudes and intentions, and by gynecological and illness experiences, different family support and decision-making processes, expectations about results and changes in nutritional habits (Bayked et al.).

The fifth contribution provides an in-depth analysis of the fact that open defecation is significantly practiced also by households with latrines. The mere presence of a latrine in itself is not a sufficient factor to reduce the practice of open defecation. Such practice increases steadily the transmission of microorganisms that cause several infections, for which children are the most vulnerable group (Ismail et al.).

The following article documents that disparities in knowledge on nutrition are related to gender, level of education and depend on the possibilities of access to nutritional information. Targeted interventions focused on improving nutritional literacy among students need to be developed as part of health promotion efforts in a comprehensive perspective (Edin et al.).

The seventh article reports how significant disparities in access to basic sanitation services exist between rural and urban households in Ethiopia. Key factors contributing to these disparities include the age of the household head, education level, family size, region of residence, and water source proximity (Keleb et al.).

The last article highlights the critical role of minimum dietary diversity in addressing undernutrition among children aged 6–23 months in Ethiopia. Targeted interventions focused on promoting diversified diets and better quality nutrients for children, together with improving access to essential health services, will mitigate the burden of undernutrition and can ensure greater wellbeing for Ethiopia's children and youth (Shibeshi et al.).

The translation into practice of paradigms of healthy lifestyles and nutrition (6) is possible considering profile and local availability of resources, acceptability, and verified substitutes (7).
